# Impact of diet and genes on murine autoimmune pancreatitis

**DOI:** 10.1111/jcmm.15540

**Published:** 2020-07-08

**Authors:** Robert Jaster, Yask Gupta, Sarah Rohde, Luise Ehlers, Horst Nizze, Artem Vorobyev, Ralf J. Ludwig, Saleh M. Ibrahim

**Affiliations:** ^1^ Department of Medicine II Division of Gastroenterology Rostock University Medical Center Rostock Germany; ^2^ Lübeck Institute of Experimental Dermatology and Center for Research on Inflammation of the Skin University of Lübeck Lübeck Germany; ^3^ Institute of Pathology Rostock University Medical Center Rostock Germany; ^4^ Department of Dermatology and Center for Research on Inflammation of the Skin University of Lübeck Lübeck Germany

**Keywords:** autoimmune pancreatitis, diet, gene‐environment interactions, *Map3k7*, MRL/MpJ mice, QTL mapping, susceptibility genes

## Abstract

The impact of environmental factors, such as diet, and the genetic basis of autoimmune pancreatitis (AIP) are largely unknown. Here, we used an experimental murine AIP model to identify the contribution of diet to AIP development, as well as to fine‐map AIP‐associated genes in outbred mice prone to develop the disease. For this purpose, we fed mice of an autoimmune‐prone intercross line (AIL) three different diets (control, calorie‐reduced and western diet) for 6 months, at which point the mice were genotyped and phenotyped for AIP. Overall, 269 out of 734 mice (36.6%) developed AIP with signs of parenchymal destruction, equally affecting mice of both sexes. AIP prevalence and severity were reduced by approximately 50% in mice held under caloric restriction compared to those fed control or western diet. We identified a quantitative trait locus (QTL) on chromosome 4 to be associated with AIP, which is located within a previously reported QTL. This association does not change when considering diet or sex as an additional variable for the mapping. Using whole‐genome sequences of the AIL founder strains, we resolved this QTL to a single candidate gene, namely *Map3k7*. Expression of *Map3k7* was largely restricted to islet cells as well as lymphocytes found in the exocrine pancreas of mice with AIP. Our studies suggest a major impact of diet on AIP. Furthermore, we identify *Map3k7* as a novel susceptibility gene for experimental AIP. Both findings warrant clinical translation.

## 1 INTRODUCTION

Autoimmune pancreatitis (AIP) is a rare but clinically relevant form of chronic pancreatitis (CP). AIP patients may present with obstructive jaundice due to the formation of inflammatory pseudotumours, a finding that may evoke the differential diagnosis of pancreatic cancer and result in surgical treatment. Correct diagnosis is of particular importance since AIP patients, in contrast to patients with other forms of CP, usually respond well to steroid treatment.^1^, ^2^ The current concept of AIP pathophysiology differentiates two subtypes of AIP, AIP type 1 and type 2, which are distinguished by their pathogenesis: AIP type 1 is the pancreatic manifestation of IgG4‐related disease and is characterized by dense infiltrates of IgG4‐positive plasma cells. In AIP type 2, which is frequently associated with inflammatory bowel disease, granulocytic epithelial lesions are the key pathognomonic finding, whereas IgG4‐expressing plasma cells are lacking. Common histological features of both subtypes are a periductal fibrosis and the presence of periductal lymphoplasmacytic infiltrates.^1^, ^2^, ^3^ AIP shares with other autoimmune diseases basic immunological characteristics, such as presence of autoantibodies and involvement of autoreactive T cells. Furthermore, adoptive transfer of splenic leucocyte subpopulations (specifically, CD44^high^ memory T cells) from MRL/MpJ mice with AIP into healthy mice induces AIP in the recipients.^4^

Environmental and nutritional influences are well‐established key factors in the pathogenesis of pancreatitis. Of note, chronic alcohol abuse represents the most common cause of CP in general, and hypertriglyceridemia is associated with an increased risk of acute pancreatitis.^5^ The role of such factors in the development of AIP has, however, not been systematically studied yet. Therefore, we here also addressed the contribution of diet, as an every‐day environmental factor, on AIP development.

Regarding genetic associations, genome‐wide association studies (GWAS) and candidate gene‐based approaches have identified several susceptibility loci for human AIP (largely, type 1), including HLA DRB1*04:05‐DQB1*04:01,^6^ *FCRL3*,^7^ *CTLA4* ^8^, ^9^ and *KCNA3*.^10^ Targeted sequencing has suggested additional genetic associations of AIP, specifically *CACNA1S*, *SMAD7*, *TOP1* ^11^ and *CALCB*.^12^ Noteworthy, specific *PRSS1* mutations (*PRSS1*_IVS 2+56_60 delCCCAG and *PRSS1*_p.Leu81Met) that were suggested to cause ectopic trypsinogen activation have also been implicated into the pathogenesis of human AIP type 1.^13^, ^14^ Further insights into the genetic architecture of AIP have been obtained using the MRL/MpJ mouse AIP model. At an age of approximately 6 months, predominantly female MRL/MpJ mice spontaneously develop AIP that resembles important histopathological features of human AIP.^15^, ^16^ Application of polyinosinic:polycytidylic acid (poly I:C) further accelerates and enhances the disease.^17^, ^18^ Previously, we generated an autoimmune‐prone advanced intercross mouse (AIL) line, to study the impact of genetics and diet on complex (inflammatory disease) traits in the mouse, including AIP.^19^ In brief, 3 parental mouse strains that spontaneously develop different autoimmune diseases—MRL/MpJ (AIP), NZM2410/J (lupus) and BXD2/TyJ (arthritis)—and CAST/EIJ mice, which are not prone to any autoimmune disease, were subjected to crossbreeding.^20^ These AIL mice have been used for QTL mapping of several complex traits, including murine AIP.^19^, ^20^ Regarding AIP, 5 associated QTL were identified, located on chromosomes 2, 4 (two QTLs), 5 and 6.^20^ The identified QTL, however, encompassed numerous genes, due to the fact that the study was performed in mice of the 4th generation, where relatively few crossing over had occurred. To fine map these QTLs, preferably to the single gene level, we here used mice of the 15th, 18th‐20th generation. In addition, we took advantage of recently published whole‐genome sequences (WGS) of the AIL founder strains ^19^ to fine map the genetic associations of AIP.

## 2 MATERIALS AND METHODS

### 2.1 Animals

**FIGURE 1:**
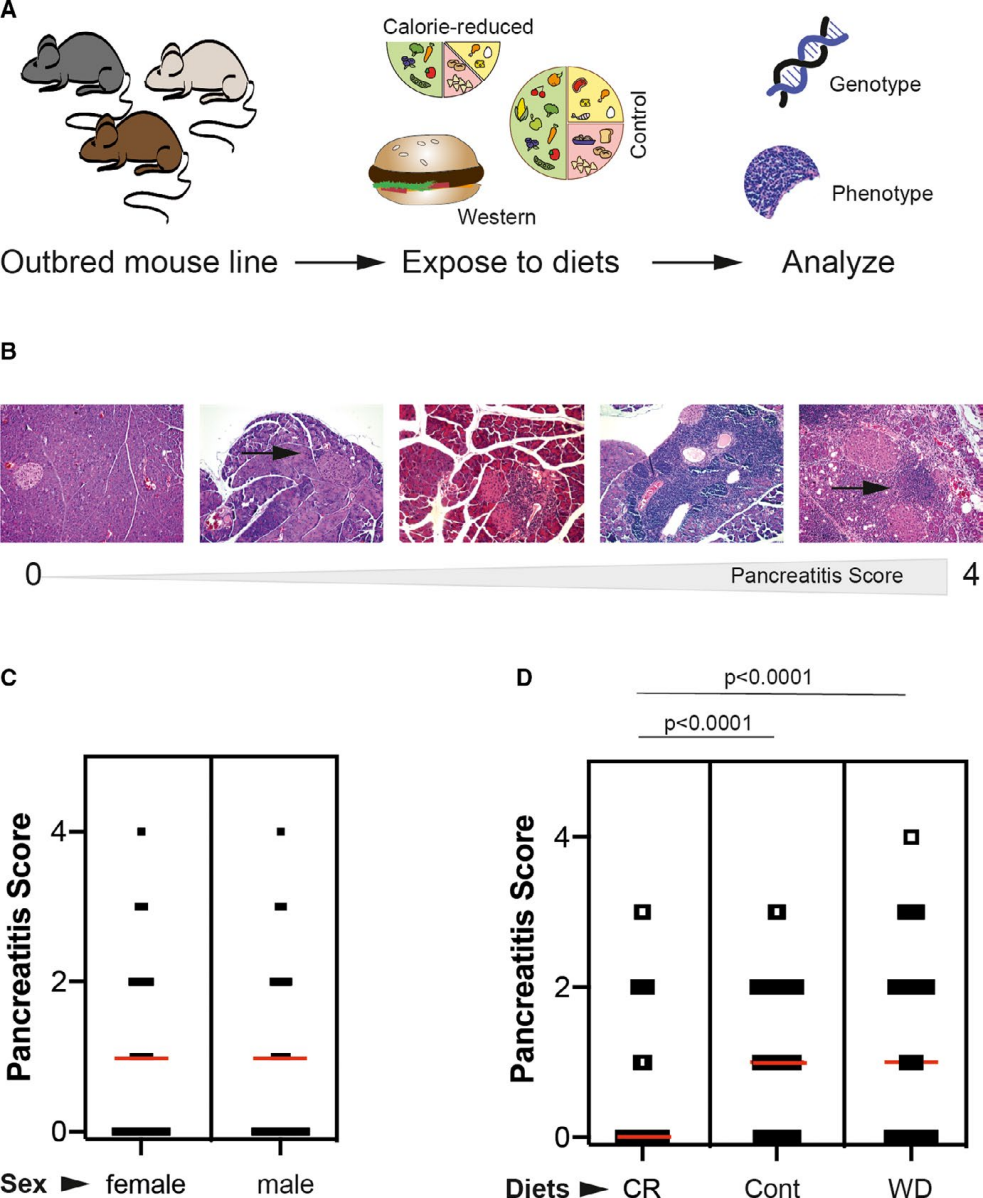
Diet modulates the spontaneous development of autoimmune pancreatitis (AIP) in an advanced intercross outbred mouse line (AIL) (A) A total of 734 outbred AIL mice were fed 3 different diets (caloric restriction, control or western diet) for 6 months. Thereafter, mice were genotyped and H&E‐stained sections from the pancreata were evaluated for the presence and (if present) severity of AIP. (B) Pancreatic sections were stained with H&E and subjected to the evaluation of pathological changes on a semi‐quantitative scale from 0 to 4. Stage 0: healthy; stage 1: small infiltrate of mononuclear cells (arrow); stage 2: large periductal focus of mononuclear cells; beginning parenchymal destruction; stage 3: severe inflammation and more extended parenchymal destruction; and stage 4: organ‐wide inflammation; large‐scale destruction of acini and partial replacement by adipose and fibrotic tissue. (C) When stratified for sex, no difference in the AIP severity was noted. Graph shows all individual values as dots, whereas the red line indicates the median. Statistical analysis was performed using rank‐sum test. (D) By contrast, stratification for the different diets showed that mice fed calorie‐reduced diet had a significant lower AIP severity compared to control and western diet‐fed mice. Graph shows all individual values as dots, whereas the red line indicates the median. Statistical analysis was performed using one‐way ANOVA (*P* < 0.0001) with Tukey's multiple comparison as post‐test

MRL/MpJ, NZM2410/J, BXD2/TyJ and CAST/EiJ parental mouse strains were intercrossed at an equal strain and sex distribution as described ^4^, ^19^, ^20^ to generate an advanced autoimmune‐prone intercross line (AIL). As previously reported, AIL mice develop AIP,^20^ which is most likely due to the inclusion of the AIP‐susceptible MRL/MpJ mice.^16^, ^18^, ^19^, ^20^ For the mapping study herein, mice of the 15th, 18th‐20th generation were used. To study the impact of diet on AIP, after weaning, AIL mice were randomly allocated to one of the following diets: control mouse chow, caloric restriction and western diet, as described elsewhere.^19^ Regarding randomization: Offspring mice were transferred into separate cages after weaning at 3‐4 weeks. Each cage contained mice of either gender and was randomly allocated at a 1:1:1 to one of the three different diets: control mouse chow, caloric restriction and western diet (all mice of one cage received the same diet, which was selected at random). At the age of 6 months, a skin biopsy (for genotyping) and the pancreas (for histological analysis) were obtained (Figure 1A). A total of 734 mice lived until that age; of these, 461 were female and 273 were male. A total of 84, 276 or 101 female mice were in the caloric restriction, control chow or western diet arms, respectively. For males, this amounted to 79, 140 or 54 mice (Table 1). Unequal sample size distribution is due to the cage‐wise randomization and the death of mice during the 6‐month observation period. Mice were held under specific pathogen‐free conditions at 12‐hour light/dark cycle at the animal facility of the University of Lübeck, Germany. Animal experiments were conducted according to the European Community rules for animal care, approved by the respective governmental administration (Ministry for Energy, Agriculture, the Environment and Rural Areas, file number 27‐2/13) and performed by certified personnel.

### 2.2 Genotyping and QTL mapping

The tips of the tails of AIL mice were collected during mouse sampling at month 6 and subjected to the isolation of genomic DNA as reported.^19^ In brief, the DNeasy Blood & Tissue Kit (Qiagen GmbH, Hilden, Germany) was employed according to the manufacturer's protocol, and DNA samples were stored at −20°C until further use. DNA samples from 734 mice were analysed by MegaMUGA genotyping array covering 77 800 markers throughout the mouse genome.^21^ Genotyping was performed at Neogen/GeneSeek (Lincoln, NE, USA). Non‐informative SNPs were filtered out using plink and applying the following criteria: minor allele frequency (maf) >0.05, missing genotype probability <0.1 and common homozygous SNPs among the founders. This approach yielded 55,458 informative SNPs for further downstream analysis.^22^ The probabilistic reconstruction of AIL mouse genome in term of four founder strains was performed using the R package of HAPPY^23^, ^24^ as described before.^19^ Briefly, the posterior probability that each mouse was in one of the four possible genotype states was estimated employing a hidden Markov model (HMM), and probabilities were converted to Rqtl2 object in R. Subsequently, a chromosome‐wise kinship matrix, which represents intra‐individual relationship, was calculated using 'calc_kinship' function with leaving out one chromosome at a time (R/qtl2 R package).^25^ We estimated heritability of AIP using 'est_herit' function from the same R package, that is R/qtl2. For additive model, LOD scores were calculated by linear mixed model in which AIP score was regressed to posterior probabilities with sex and diet as additive covariate and kinship as a random effect (scan1 function from Rqtl2). For QTL interaction model, covariates diet and sex were considered interactive covariates rather than additive covariate within the same function. Genome‐wide and suggestive thresholds were estimated by traditional permutation (1000)‐based approach at 5% and 10% threshold. The confidence interval for a QTL was described by 1.5 LOD drop within 10 Mb from the peak SNP. To further fine map the QTL, WGS data from founder strains (variant call file format; vcf) were downloaded from database.^19^ The annotation of SNPs and Indels within this file was performed using ENSEMBL VEP web server.^26^ The genomic region plots for the QTL were visualized and created using R ggplot2 ^27^ and UCSC Genome Browser.^28^ This analytical pipeline has been recently validated.^9^

### 2.3 Histology and immunohistochemistry

Development of spontaneous AIP was assessed based on pancreatic histopathology. Therefore, paraffin‐embedded pancreatic sections (4 µm thick) were stained with haematoxylin and eosin (H&E), and subjected to the evaluation of pancreatic lesions on a semi‐quantitative scale from 0 to 4 as described before.^16^, ^18^, ^20^ Briefly, the stages were defined as follows: 0, no pathological findings; 1, minimal focal infiltration of periductal tissue with mononuclear cells but lack of parenchymal destruction; 2, presence of larger periductal foci of mononuclear cells along with beginning parenchymal destruction; 3, severe and multifocal periductal inflammation together with more extended parenchymal destruction; and 4, comprehensive infiltration of pancreatic tissue with mononuclear cells, large‐scale destruction of acini and (partial) replacement by adipose or fibrotic tissue (Figure 1B). All samples were assessed by microscopic analysis of at least three tissue sections per sample by two independent investigators and blinded before evaluation.

Immunohistochemistry was performed on 4 µm thick paraffin‐embedded pancreatic sections. The sections were deparaffinized and subsequently stained using the ImmPRESS—alkaline phosphatase detection system according to the instructions of the manufacturer (Vector Laboratories, Burlingame, CA, USA). For staining of MAP3K7, a mouse protein‐specific polyclonal antibody from Biorbyt, Cambridge, UK (catalogue number: orb32004) was employed. The slides were then counterstained with Mayer's hemalum solution, dehydrated by two short incubations in ethanol and xylene each and embedded in Pertex (MEDITE, Burgdorf, Germany).

### 2.4 Statistical analysis

Unless stated otherwise, data were analysed employing IBM SPSS Statistics V25.0 and are presented as mean values ± SEM. Statistical tests are detailed in the figure legends. *P* values of < 0.05 were considered statistically significant. We used Dsquare function from modEvA R package to estimate proportion of variance explained by diet (4.7%) and sex (0.3%) for pancreatitis score.

## 3 RESULTS

### 3.1 Caloric restriction reduces the prevalence and clinical severity of autoimmune pancreatitis

At 6 months, 428 out of 734 mice (58.3%) presented with lymphocytic infiltrates in the exocrine pancreas. A total of 269 animals (36.6%) displayed signs of parenchymal destruction and were classified as AIP stage 2 or greater. Both sexes were similarly affected by AIP (Figure 1C). Prevalence of AIP stage 2 or greater amounted to 38.5% in female and 33.4% in male mice, and average AIP scores were 1.1 ± 0.4 or 0.9 ± 0.4 for female or male mice, respectively. AIP scores also showed no differences between both sexes when calculation was performed separately within each dietary group. When comparing AIP prevalence among the different diets, 71% mice under caloric restriction were free of any AIP, whereas an AIP score of 0 was only observed in 30% and 41% of the mice fed control and western diet, respectively (*P* < 0.05; chi‐squared test). Corresponding findings were made regarding AIP severity: In mice held at caloric restriction, the histological AIP score was 0.5 ± 0.03; whereas the score reached 1.1 ± 0.03 or 1.2 ± 0.04 in mice fed control or western diet, respectively (Figure 1D). Strikingly, cases with parenchymal destruction (stages 2‐4) were much less common in mice with caloric restriction than in animals on control chow or western diet (Table 1). Taken together, these findings point towards a protective role of caloric restriction on the development and on the progression of murine AIP. The principle effects of diets on the average scores were the same in females and males (data not shown).

**TABLE 1:** Pancreatic phenotypes of AIL mice. A total of 734 mice (461 females and 273 males, all from G15‐19) were fed the indicated diets for 6 months, before phenotyping was performed by assessing pancreatic histopathology on a semi‐quantitative scale from 0 (healthy pancreas) to 4 (most severe AIP). The unequal sex distribution is due to (i) the sex ratio at birth (which was in favour of females), and (ii) a higher mortality rate in male mice during the 6 months of the experiment

Sex	Diet	Score pancreas					Scores 2‐4 (%)	Scores 0‐1 (%)
		0	1	2	3	4		
Female	Caloric restriction	60	7	14	3	0	20.2	79.8
	Control chow	75	87	107	7	0	41.3	58.7
	Western diet	43	11	32	14	1	46.5	53.5
Male	Caloric restriction	56	6	14	3	0	21.5	78.5
	Control chow	51	43	41	5	0	32.9	67.1
	Western diet	21	5	21	7	0	51.9	48.1
Both	Caloric restriction	116	13	28	6	0	20.9	79.1
	Control chow	126	130	148	12	0	38.5	61.5
	Western diet	64	16	53	21	1	48.4	51.6

### 3.2 Mitogen‐activated protein kinase kinase kinase 7 (Map3k7) is associated with experimental autoimmune pancreatitis

**FIGURE 2:**
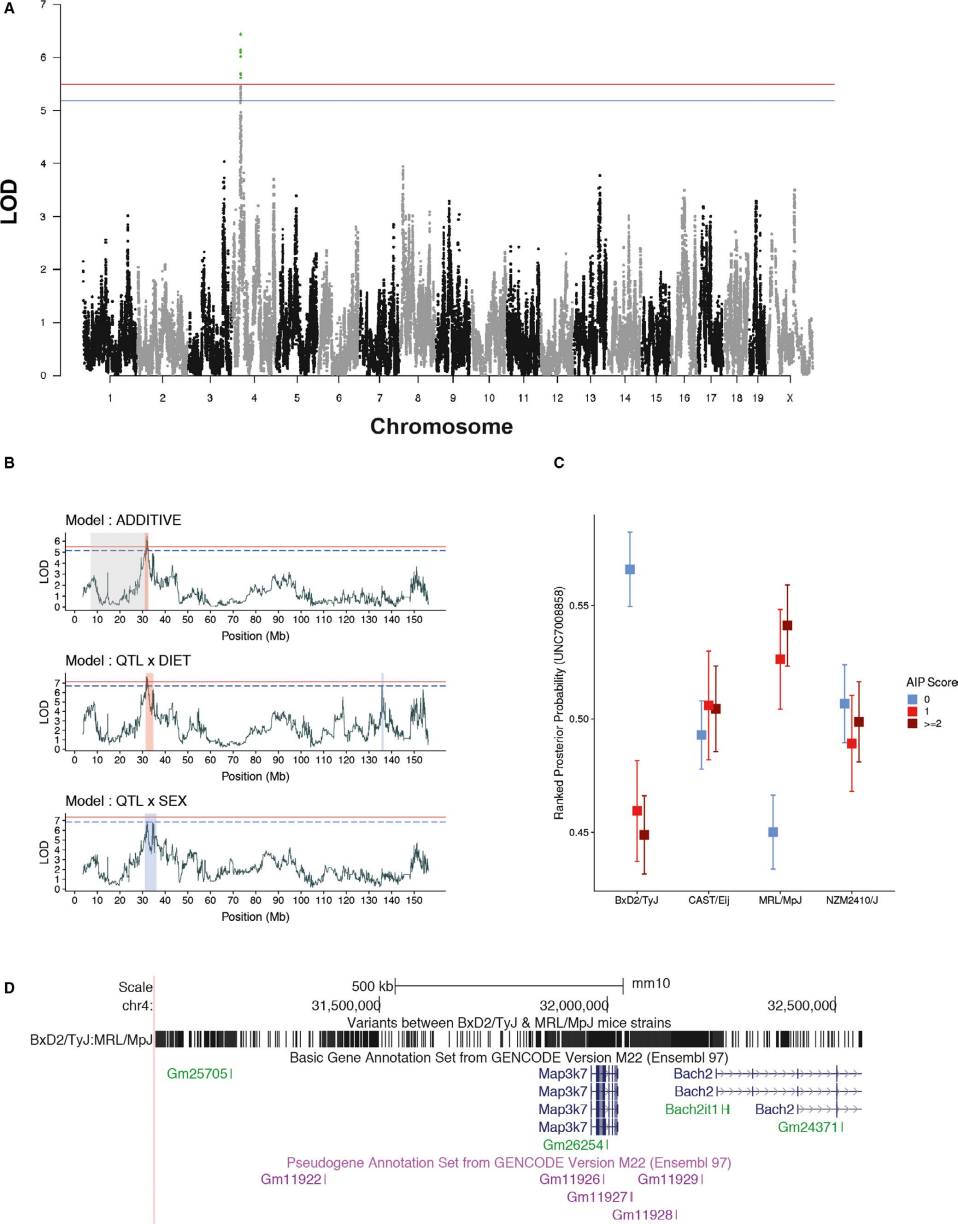
*Map3k7* is associated with experimental autoimmune pancreatitis (A) Manhattan plot for mapping of quantitative trait loci (QTLs) for murine autoimmune pancreatitis (AIP9. The plot shows chromosomes on *x*‐axis and LOD (log of odd ratios) for each SNP (grey and black dots) on *y*‐axis. LOD scores were calculated by regressing histological pancreatitis score with the estimated genotype posterior probability. Sex, diet and first principle component of kinship matrix were considered as additive covariate loci. Logarithmic p‐values are plotted against genome location. The red line represents the genome‐wide threshold (*P* < 0.05), and the blue line shows the suggestive threshold (*P* < 0.1) evaluated after 1000 permutations for histological pancreatitis score. (B) The scatter line plot shows LOD scores across chromosome 4 for three models (ie additive, interactive diet and sex). Use of either one of the interactive variables ('diet' or 'sex') considers the variability introduced by either of the variables, allowing to detect gene‐diet and gene‐sex interactions. The shaded region within the plot described confidence interval in Mb for each model (grey = AIP2, orange = genome‐wide and blue = chromosome‐wide). (C) The box plot shows ranked haplotype posterior probabilities (derived from happy R package using HMM) for Peak SNP (UNC7008858) across the four founder strains, when stratified for different AIP score. (D) The figure shows the fine‐mapped AIP2 loci (using UCSC browser) on chromosome 4 and genes within this loci. The figure also shows all the SNPs and Indels (derived from WGS) different between BxD2/TyJ and MRL/MpJ, as Peak SNP from these strains was negatively and positively correlated with AIP score in previous figure

Next, we aimed to fine map previously reported QTLs of AIP,^20^ possibly identify so far unreported AIP‐associated genes, and evaluate the impact of gene‐diet and gene‐sex interactions in AIP. For fine mapping, we continuously intercrossed AIL mice leading to smaller confidence intervals of QTL due to the meiotic crossing over. In the 15th, 18th‐20th AIL mouse generation, 734 mice were genotyped and phenotyped for AIP severity. Considering diet and sex as additive variables, we identified a QTL at the genome‐wide significance level on chromosome 4 (LOD = 6.44, Confidence interval (CI) = 31‐32.6 Mb, Figure 2A‐B), which was located within the previously identified QTL for AIP (Table 2). We next considered diet or sex as interactive covariates for the QTL mapping, allowing to identify gene‐diet and gene‐sex interaction: The QTL on chromosome 4 was confirmed at the genome‐wide significance level (LOD = 7.68, CI = 31.39‐34.8 Mb) when diet was taken as an interactive variable. When using sex as an interactive variable, the QTL was significant at the suggestive level (LOD = 6.89, CI = 31‐36.1 Mb, Figure 2B). We also mapped a suggestive QTL interacting with diet on chromosome 4 (LOD = 6.76) at CI of 135.5‐136.4 Mb (Table 2), suggesting that diet can potentially unmask novel genetic associations also in AIP. In addition, 6.8% or 0.46% of AIP phenotypic variance are explained by diet or sex, respectively.

**TABLE 2:** Pancreatitis Score QTLs identified by fine mapping. Genome‐wide threshold for LOD (0.05), suggestive threshold (0.1). In addition, 6.8% or 0.46% of AIP phenotypic variance are explained by diet or sex, respectively

Chr	CI (Mb)	Peak SNP	Peak Pos (Mb)	Pheno var %	LOD	Model	Significance
4	31‐32.56	UNC7008858	31.94	3.96	6.44	Additive	Genome
4	31.39‐34.8	UNC7008858	31.94	4.70	7.68	QTL x Diet	Genome
4	135.55‐136.48	UNC8290092	135.77	4.15	6.76	QTL x Diet	Suggestive
4	31‐36.12	UNC7008858	31.94	4.23	6.89	QTL x Sex	Suggestive

Abbreviations: Chr, chromosome; CI, confidence interval; pheno var, phenotypic variability.

Still, the fine‐mapped QTL, located on chromosome 4, contained several genes. Hence, for further fine mapping, we first correlated (Spearman rank correlation) the posterior probability of the peak SNP (UNC7008858) for the individual founder strains to the AIP score. Here, BxD2/TyJ and MRL/MpJ strains negatively correlated (*ρ* = −0.18, *P* < 0.01) and positively (*ρ* = 0.14, *P* < 0.01) with AIP score. The other two strains CAST/EiJ (*ρ* = 0.02, *P* = 0.57) and NZM2410/J (*ρ* = 0.0007, *P* = 0.98) showed no correlation with the AIP score (Figure 2C). Hence, as indicated by the haplotype effects, the genetic association of the AIP score is due to the genetic differences between the BxD2/TyJ and MRL/MpJ strains within this locus. Next, we used the recently published WGS of the 4 AIL founder strains ^19^ to identify polymorphisms (SNPs and Indels) discriminating between BxD2/TyJ and MRL/MpJ strains. We identified 2,994 variants between the two strains among which most of the variants were either intergenic regions (38%), intronic (36%) or located within non‐coding transcripts without any predicted severe consequences. For protein‐coding genes, we did not identify any non‐synonymous variants with severe consequences. However, we detected multiple variants on predicted upstream/downstream region and importantly in 3’ UTR region of the full‐length protein‐coding gene *Map3k7*, whereas for the partial protein‐coding gene *Bach2* variants in predicted upstream region were found (Figure 2D). The importance of variants in 3’ UTR has been established previously. Hence, we identify *Map3k7*, also known as *transforming growth factor‐beta‐activated kinase 1* (*Tak1*), spans from 31,96 to 32,02 Mb, to be potentially associated with AIP.

### 3.3 Map3k7 is expressed by infiltrating mononuclear cells of mice with experimental autoimmune pancreatitis

**FIGURE 3:**
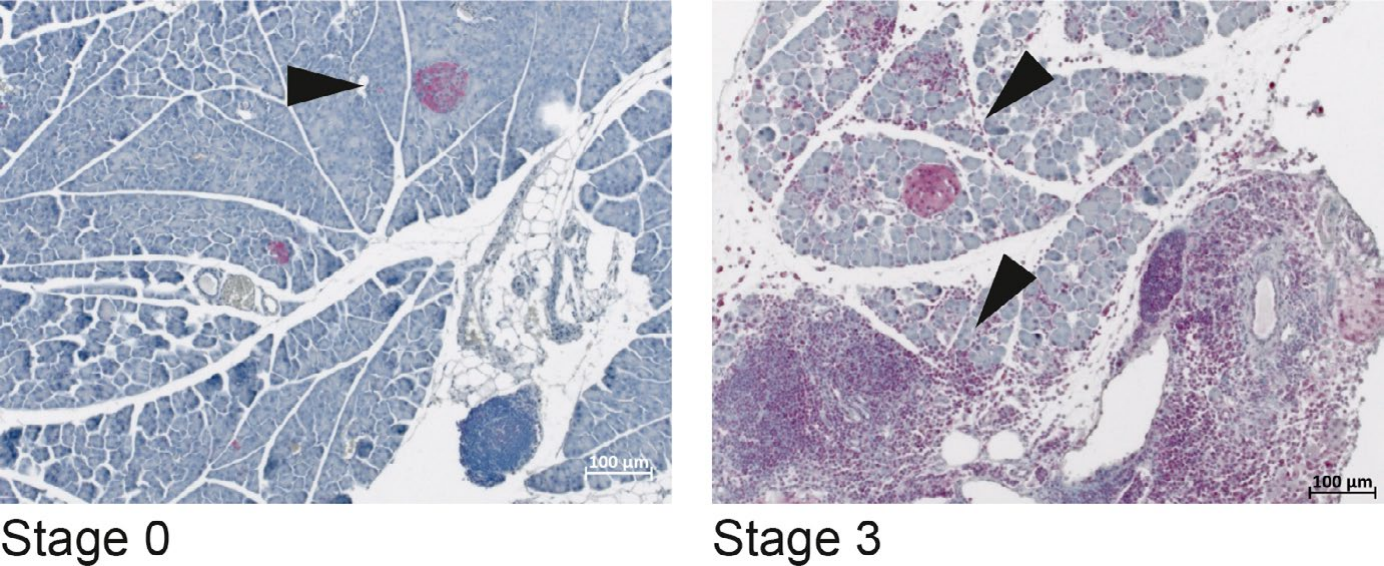
*MAP3K7* is expressed in the lymphocyte infiltrates of experimental autoimmune pancreatitis Pancreatic tissues from mice with AIP stage 0 (healthy) and stage 3 (severe AIP) were stained with anti‐MAP3K7. Arrows point to positively stained pancreatic islets and organ‐infiltrating mononuclear cells, respectively. The photographs are representative for n ≥ 5 mice per group

To obtain insights into a potential contribution of *Map3k7*, we studied its protein expression in the context of murine AIP. In healthy pancreata, MAP3K7 protein expression was largely restricted to pancreatic islets (Figure 3). By contrast, pancreata from mice with AIP showed additional expression by the pancreas‐infiltrating mononuclear cells (Figure 3).

## 4 DISCUSSION

Autoimmune disorders are multifactorial diseases that are influenced by the interplay of environmental, for example dietary, and genetic factors.^19^ In the case of AIP, both aspects are still relatively poorly understood. We herein addressed this knowledge gap and show that diet has a vast impact on AIP development. More specifically, compared to control or western diet, caloric restriction halved both AIP prevalence and severity. Regarding the genetic AIP association, we fine‐mapped *Map3k7* as an AIP‐associated gene and demonstrate its expression in the leucocyte infiltrate within the pancreas of mice with AIP.

Our genetic studies were based on the report of five, rather large QTLs that were mapped within in AIL mice in the 4th generation,^20^ including one QTL located on chromosome 4 (termed *AIP2*). In order to obtain smaller confidence intervals of the QTLs, we took advantage of meiotic crossing overs across generations. By continued intercrossing of AIL mice for 20 generations and use of WGS data of the founder strains, we were here able to confirm and to narrow down the size of one of these QTLs, namely *AIP2*.

The herein identified QTL within the *AIP2* locus encodes for one protein‐coding gene, *Map3k7* (*Tak1*). By contrasting the genomic variations of the AIL founder strains, the variability within this QTL derived from the AIP‐prone MRL/MpJ strain, whereas the *Map3k7* allele of BXD2/TyJ mice was associated with less AIP.

The MAP3K7 protein represents an essential signalling intermediate in tumour necrosis factor, interleukin 1, and Toll‐like receptor signalling pathways. The member of the mitogen‐activated protein kinase (MAPK) kinase kinase family transmits upstream signals from the receptor complexes to the downstream MAPKs and to the NF‐κB pathway.^29^, ^30^, ^31^ Whereas *Map3k7* has not been associated with AIP previously, mutations within this gene are associated with other experimental inflammatory diseases in the mouse, including murine autoimmune myocarditis,^32^ encephalomyelitis^33^ and type 1 diabetes.^33^ In the latter two models, an inhibitor of MAP3K7/TAK1, 5Z‐7‐oxozeaenol (OZ), attenuated progression of the disease and will therefore be of interest in the context of experimental AIP as well. The mechanisms of OZ action are apparently complex and may involve impairment of dendritic cell maturation and a Th1 to Th2 cytokine shift.^34^ However, when mapping 54 physiological and pathophysiological phenotypes, including ANA production and lupus susceptibility, no association with *Map3k7* was found, pointing towards a selective role of this gene in AIP pathogenesis.^18^

In addition to *AIP2*, we previously have identified additional QTLs for AIP located on chromosomes 2, 5 and 6.^20^ These previously reported QTLs were, however, not replicated within the current study. This is likely because of the epistatic interactions or gene environmental interactions. It should also be noted that generation 4th AIL mice were housed at the University of Rostock, whereas later generations were kept at the University of Lübeck. These changes in microenvironment may have altered the association of AIP with host genetics. For example, when we introduced diet (microenvironment modulator) as an interacting covariate, the highest LOD scores on chromosome 2 (LOD = 4.07) and 5 (LOD = 5.25) were observed at 65.9 (SNP = UNC3225113) and 78.7 (SNP = UNC9531776) Mb. The respective regions fall within the confidence intervals of the previously reported QTLs on chromosome 2 (56.3‐81.9 Mb) and chromosome 5 (74.8‐96.6 Mb) but did not pass the significance threshold. These discrepancies were also reported in previous studies and should be further investigated for AIP.^19^ *AIP2* comprised a CI of 25.2 Mbp (Mbp 7.1‐32.3), contained a variety of genes and was therefore too large for the analysis of genetic traits at the level of individual genes. In contrast, the herein fine‐mapped QTL was 1.55 Mb only and contained several predicted and pseudogenes, a full‐length protein‐coding gene (*Map3k7*) and a partial protein‐coding gene (*Bach2*). The latter gene might be of interest for follow‐up studies as well since recent data suggest that *Bach2* repression is associated with clinical features of advanced CP through an increased Th17 cell‐induced inflammation,^35^ a subset of T cells that may also play a role in murine AIP.^4^

Of note, the QTL effect observed in this study (Figure 2) cannot be caused by a single SNP, as the effect (a) differs between strains CAST/EIJ + NZM2410/J (both no effect) versus BXD2/TyJ + MRL/MpJ, (b) but BXD2/TyJ + MRL/MpJ differ according to severity. The contribution of different SNPs to this complex pattern represents a point of interest for follow‐up investigations.

Using the same autoimmune‐prone AIL as in this study, we have recently shown that diet substantially contributes to the variability of complex traits and, in addition, may unmask novel susceptibility QTLs.^19^ Strikingly, caloric restriction alone was sufficient to overcome the genetic susceptibility of the mice to develop lupus. This observation prompted us to ask whether diet might also influence the development of autoimmune pancreatitis in genetically susceptible mice. The results of this study show that, compared to control chow or western diet, long‐term caloric restriction roughly halved the rate of AIP with parenchymal destruction in our model. Western diet, on the other hand, did not significantly affect the course of the disease, although we noticed a tendency towards more severe stages of AIP (Table 1). The effects of diet were observed in both sexes, which in this study did also not significantly differ with respect to the overall occurrence of AIP. To the best of our knowledge, this is the first study to show an effect of diet on the development of AIP in either an experimental model or AIP patients. In a rat model of non‐insulin‐dependent diabetes with obesity, WBN/Kob‐fatty rats, caloric restriction was found to be associated with less severe signs of spontaneous CP and specifically diminished interlobular, intra‐lobular and intra‐islet fibrosis. These findings, however, could be attributed to a unique genetic background, the homozygous presence of the *fa* allele of the leptin receptor gene, and are also not directly linked to AIP.^36^ Apart from AIP, growing evidence from experimental and clinical studies suggests that caloric restriction and fasting‐mimicking diets may positively impact autoimmune diseases such as systemic lupus erythematosus (SLE), rheumatoid arthritis, multiple sclerosis and autoimmune diabetes.^19^, ^37^, ^38^ In addition, we recently demonstrated that caloric restriction confers complete protection from the development of lupus nephritis in NZM2410/J mice, whereas mice fed western diet showed an accelerated and aggravated clinical phenotype. Interestingly diet‐induced changes in the intestinal micro‐ and mycobiome preceded clinical onset, indicating that the protective effects of diet may be modulated by changes in the intestinal microbiome.^19^ Although it is well known that the type and levels of nutrients can influence the generation, survival and function of lymphocytes, the mechanistic links between caloric restriction and autoimmunity are far from being understood. Given the potential clinical implications of dietary interventions, our results encourage further studies in the context of AIP.

In summary, this investigation has identified a novel susceptibility gene of murine AIP, *Map3k7*, and a potent protective environmental factor, dietary restriction. As the MAP3K7 protein is in principle druggable^39^ and dietary interventions are possible, these findings may shape the future management of AIP patient.

## CONFLICT OF INTEREST

None to declare.

## AUTHOR CONTRIBUTION

**Robert Jaster:** Conceptualization (lead); Data curation (equal); Formal analysis (equal); Funding acquisition (equal); Investigation (equal); Methodology (equal); Project administration (lead); Resources (equal); Supervision (equal); Validation (equal); Visualization (equal); Writing‐original draft (lead); Writing‐review & editing (lead). **Yask Gupta:** Data curation (lead); Formal analysis (supporting); Methodology (equal); Validation (supporting); Visualization (supporting); Writing‐original draft (supporting); Writing‐review & editing (supporting). **Sarah Rohde:** Investigation (equal); Methodology (equal); Validation (supporting); Writing‐original draft (supporting); Writing‐review & editing (supporting). **Luise Ehlers:** Investigation (equal); Methodology (supporting); Validation (supporting); Writing‐original draft (supporting); Writing‐review & editing (supporting). **Horst Nizze:** Investigation (equal); Methodology (supporting); Writing‐original draft (supporting); Writing‐review & editing (supporting). **Artem Vorobyev:** Investigation (equal); Methodology (supporting); Validation (equal); Writing‐original draft (supporting); Writing‐review & editing (supporting). **Ralf J Ludwig:** Conceptualization (supporting); Data curation (supporting); Formal analysis (supporting); Funding acquisition (supporting); Investigation (supporting); Methodology (supporting); Resources (supporting); Supervision (equal); Validation (equal); Visualization (lead); Writing‐original draft (equal); Writing‐review & editing (equal). **Saleh Ibrahim:** Conceptualization (equal); Data curation (supporting); Formal analysis (equal); Funding acquisition (equal); Investigation (supporting); Methodology (supporting); Resources (equal); Supervision (equal); Validation (equal); Visualization (supporting); Writing‐original draft (supporting); Writing‐review & editing (supporting).

## Data Availability

The data that support the findings of this study are available from the corresponding author upon reasonable request.
